# The C9orf72 expansion is associated with accelerated respiratory function decline in a large Amyotrophic Lateral Sclerosis cohort

**DOI:** 10.12688/hrbopenres.12940.1

**Published:** 2019-09-26

**Authors:** James Rooney, Deirdre Murray, Anna Campion, Hannah Moloney, Rachel Tattersall, Mark Doherty, Michaela Hammond, Mark Heverin, Russell McLaughlin, Orla Hardiman

**Affiliations:** 1 Academic Unit of Neurology, Trinity Biomedical Sciences Institute, Trinity College Dublin, Dublin, Ireland; 2 Beaumont Hospital, Dublin, Ireland; 3 Smurfit Institute of Genetics, Trinity College Dublin, Dublin, Ireland

**Keywords:** amyotrophic lateral sclerosis, ALS, respiratory function, C9orf72, disease progression, prognosis

## Abstract

**Introduction**: The *C9orf72* hexanucleotide repeat expansion is causal in amyotrophic lateral sclerosis (ALS) and has a negative effect on prognosis. The *C9orf72* repeat expansion has been associated with an accelerated deterioration of respiratory function and survival in a cohort of 372 Portuguese patients.

**Methods**: Cases presenting to the Irish ALS clinic with both longitudinal occluded sniff nasal inspiratory pressure (SNIP) and *C9orf72* testing were including in the study. Clinical variables and survival characteristics of these patients were collected. Joint longitudinal and time to event models were constructed to explore the longitudinal characteristics of the cohort by *C9orf72* status.

**Results**: In total, 630 cases were included, of which 58 (9.2%) carried the *C9orf72* repeat expansion. Plots of the longitudinal trend after joint modelling revealed that those carrying the expansion had worse respiratory function throughout the course of their disease than those without. The ALS Functional Rating Scale-revised (ALSFRS-R) respiratory sub-score did not distinguish *C9orf72* normal from expanded cases. Furthermore, modelling by site of onset and gender sub-groups revealed that this difference was greatest in male spinal onset cases. Joint models further indicated that occluded SNIP values were of prognostic importance.

**Conclusions**: Our results confirm findings from Portugal that the *C9orf72* repeat expansion is associated with accelerated respiratory function decline. Analysis via joint models indicate that respiratory function is of prognostic importance and may explain previous observations of poorer prognosis in male spinal onset patients carrying the *C9orf72* expansion.

## Introduction

The *C9orf72* hexanucleotide repeat expansion has been causally linked to amyotrophic lateral sclerosis (ALS) ^1, 2^ and frontotemporal dementia (FTD) ^3^. The *C9orf72* expansion accounts for up to 10% of those with ALS and 25% of FTD in populations of northern European extraction ^4^. It is associated with a number of distinctive features clinically, namely, earlier disease onset, cognitive and behavioural impairment, distinct neuroimaging changes, family history of neurodegeneration, and decreased survival relative to patients lacking the *C9orf72* expansion ^5– 12^. In 2016 we observed across five European cohorts that the negative prognosis associated with carriage of the *C9orf72* expansion is most pronounced in male patients with spinal onset ALS ^13^.

Decline in respiratory function is one of the most serious symptoms of ALS and respiratory failure is the primary cause of death in most cases; therefore, there is much interest in accurate measurement of respiratory function. We have characterised the longitudinal respiratory decline of 797 ALS and 39 primary lateral sclerosis (PLS) patients from Ireland using the occluded sniff nasal inspiratory pressure (SNIP) respiratory strength measure ^14^. The SNIP is a widely used tool that correlates well with diaphragmatic strength and is considered reliable and reproducible in ALS patients ^15^.

Recently, it has been reported that the *C9orf72* repeat expansion is associated with an accelerated deterioration of respiratory function and survival in a cohort of 372 Portuguese patients ^16^. Respiratory function was assessed with the ALSFRS-R respiratory sub-score (ALSFRS-R _resp_) and the predicted value of forced vital capacity (%FVC). It was found that %FVC declined significantly faster in patients carrying the *C9orf72* expansion compared to those without (P = 0.01), while in Cox models, the *C9orf72* expansion was associated with poorer survival (P = 0.002) ^16^. The ALSFRS-R _resp_ score was not found to be associated with *C9orf72*.

In this study, we aim to confirm the association of *C9orf72* with ** accelerated respiratory decline and prognosis in an Irish ALS cohort ** using joint longitudinal and time to event models (referred to as joint models for the rest of this manuscript). Furthermore, we aim to explore respiratory function in *C9orf72* by gender and by site of onset subgroups analogous to those we previously characterised to have differential associations with survival in male spinal onset ALS patients ^13^.

## Methods

### Ethical statement

The Irish ALS Register complies with the Irish Data Protection Acts 1988-2018 and has been approved by the Beaumont Hospital Ethics Committee (05/49). Written consent, or in cases where the disease process has affected the patients ability to write, oral consent, is obtained from all participants for inclusion on the Irish ALS register and participation in research and written documentation of consent is kept on file at the Academic Unit of Neurology, Trinity College Dublin.

### Study population

This study includes all patients with a diagnosis according to the El-Escorial criteria of spinal or bulbar onset ALS and who attended the multidisciplinary ALS clinic in Beaumont Hospital, Dublin between 01/01/2001 and 01/12/2018. The population was further limited to those who underwent respiratory assessment, and who had testing for the *C9orf72* expansion, performed using repeat-primed PCR, with a cut-off of 30 hexanucleotide repeats or above used to categorise samples as positive for the repeat expansion ^1^. The diagnosis was confirmed by the consultant neurologist (OH) and Riluzole 50mg twice daily was routinely prescribed. Patients provided informed consent for demographic and clinical data including ALSFRS-R and respiratory measurement to be recorded on the Irish ALS register. Patient’s ALSFRS-R scores were evaluated by assessors trained and certified using ENCALS standard operating procedures (ENCALS 2015). Respiratory measurement was via occluded SNIP measurement, which we described in full previously ^14^. Briefly, the preferred nostril was chosen and a nasal probe fitted. Standardized verbal instructions were provided by a trained physiotherapist and each patient completed at least 10 consecutive maximal SNIPs with the contralateral nostril occluded. The highest value for each SNIP method was recorded in cmH _2_0. Follow-up of survival status was through the regular operation of the Irish ALS register, which is carried out on a continuous basis through multiple sources including the General Register Office, www.rip.ie, and family notification of the MDT clinic staff and the IMNDA. For the current analysis, survival status was last updated at the time of data extraction from the register on 07/12/2018.

### Statistical analysis

Longitudinal models of occluded SNIP measurements were constructed as linear mixed effects multi-level models. Follow-up of cases was limited to six years for the purposes of statistical modelling as few patients (2%) survived longer than this time. Time since disease onset was included with random effects per individual and a grouped fixed effect, and occluded SNIP measurements were specified as an interaction with time. A binary term to indicate *C9orf72* expansion status was also included, interacting with time and SNIP measurements. Splines were used to allow for non-linear trend of occluded SNIP measurements over time. A delayed entry Cox proportional hazards survival model was constructed, including the important prognostic variables age at onset, diagnostic delay, bulbar onset and *C9orf72* status. The longitudinal and Cox models were then used to construct a joint model using the R package JMBayes 0.8-83 ^17^. Next, a joint model with the same explanatory variables but with the ALSFRS-R _resp_ as the dependent longitudinal variable was fitted for those participants with ALSFRS-R data. Finally, to explore the longitudinal trend of occluded SNIP measurements by *C9orf72* status in gender and site of onset subgroups, the longitudinal model of occluded SNIP was expanded to include full interaction of the *C9orf72* status, gender, site of onset and time variables, before inclusion in a new joint model. Graphs of predicted SNIP were generated from models to visualise the fitted group trends. All statistical analyses were carried out using R Statistical Software version 3.5.1 ^18^ with additional packages ^17, 19– 24^. The analysis code is provided (see *Software availability*).

## Results

In this study, 630 ALS patients with a total of 2,165 longitudinal SNIP measurements were included, of whom 58 (9.2%) carried the *C9orf72* repeat expansion. Those carrying the *C9orf72* expansion were younger (median 56.6 years) when compared to those without the expansion (median 62.0 years) but were similar in other characteristics ( Table 1). Comparison via likelihood ratio test of initial linear mixed models of *C9orf72* status versus time indicated that inclusion of spline terms improved fit (p < 0.001). Table 2 displays the hazard ratios (HRs) from the Cox proportional hazards model used for the event component of the joint model with age at onset, diagnostic delay, bulbar onset and carriage of the *C9orf72* expansion; all prognostic in the Cox model.

**Table 1.** Demographics of study patients by C9orf72 status.

	C9orf72 Normal	C9orf72 Expanded	P value
N (%)	572 (90.8)	58 (9.2)	
Male (%)	350 (61.2)	29 (50.0)	0.129
Age at onset, mean (SD)	62.0 (11.4)	56.7 (9.1)	< 0.001
Bulbar onset, N (%)	171 (29.9)	21 (36.2)	0.398
Diagnostic delay in months, median (IQR)	11.4 (6.9, 18.8)	9.0 (6.1, 19.9)	0.297
Survival time in months, median (IQR)	32.0 (21.4 – 47.2)	29.8 (19.9 – 50.4)	0.564

SD, standard deviation; IQR, interquartile range.

**Table 2.** Hazard ratios from Cox proportional hazard model and joint longitudinal and time to event models.

Variable	Cox Model	Joint Model 1	Joint Model 2
	HR (95% CI)	HR (95% CI)	HR (95% CI)
Age at onset (per year)	1.02 (1.01 – 1.03)	1.01 (1.01 – 1.03)	1.02 (1.01 – 1.03)
Diagnostic delay (per month)	0.97 (0.96 – 0.98)	0.97 (0.96 – 0.99)	0.97 (0.96 – 0.98)
Bulbar onset	1.28 (1.05 – 1.55)	0.88 (0.70 – 1.15)	0.78 (0.62 – 0.98)
*C9orf72* expansion	1.62 (1.21 – 2.15)	0.95 (0.64 – 1.38)	1.01 (0.74 – 1.42)
Longitudinal component (i.e. occluded SNIP)	_	0.96 (0.95 – 0.97)	0.96 (0.96 – 0.97)

Joint Model 1 includes a longitudinal sub-model interaction between time and *C9orf72* status; Joint Model 2 includes a longitudinal sub-model interaction between time, *C9orf72* status, site of onset and gender; SNIP, sniff nasal inspiratory pressure.

**Figure 1.**
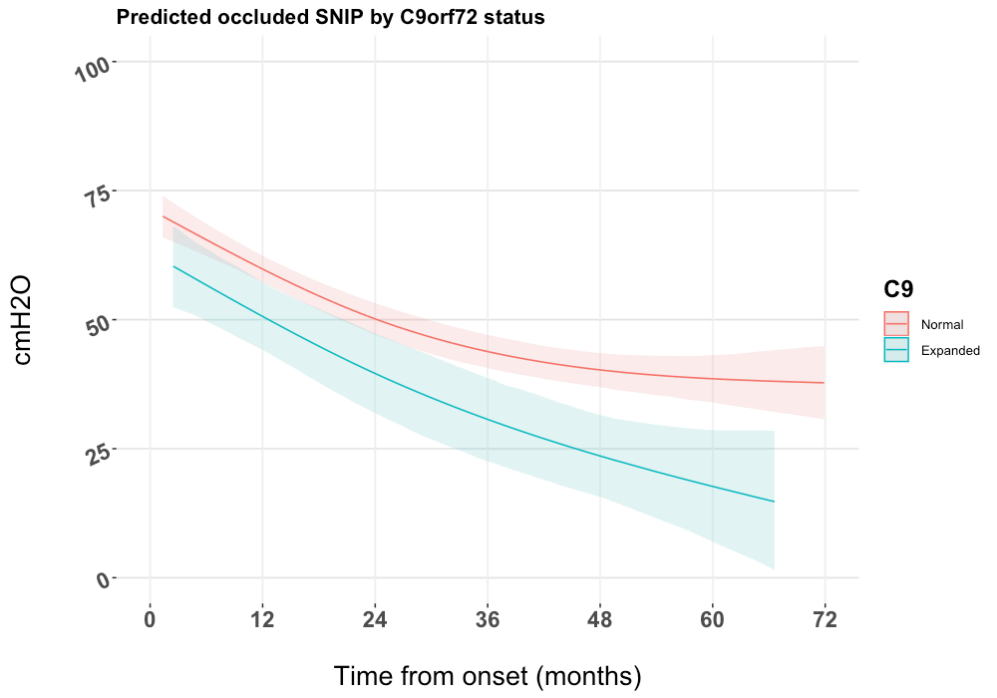
Predicted occluded sniff nasal inspiratory pressure (SNIP) by C9orf72 status generated from joint longitudinal and time to event model.

Figure 1 displays the longitudinal characteristics of the occluded SNIP measurements by *C9orf72* expansion status for all patients as modelled via joint modelling. Patients without the *C9orf72* expansion had, on average, higher scores than those carrying the *C9orf72* expansion across the complete follow-up time. The difference between groups was greater over time, particularly after three years from disease onset. Table 2 displays the hazard ratios for the Cox proportional hazards model and for the posterior estimated HRs of the survival sub-model of the joint model.

**Figure 2.**
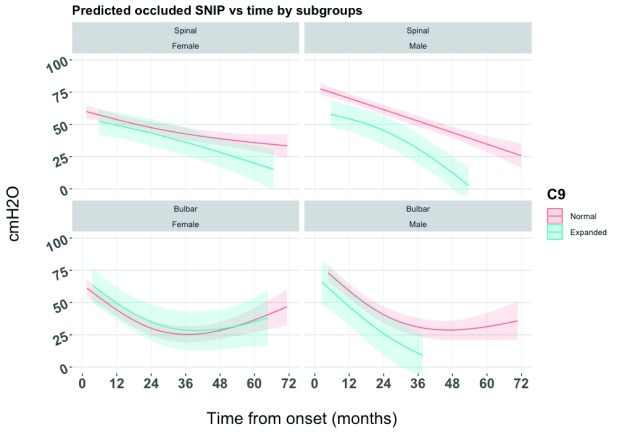
Predicted occluded sniff nasal inspiratory pressure (SNIP) by C9orf72 status generated from joint longitudinal and time to event model including interaction between time, C9orf72 status, site of onset and gender.

The exploratory model including full interaction between time, *C9orf72* status, site of onset and gender was used to generate Figure 2. The deviance information criterion (DIC) indicated a better fit for the model with interaction between time, *C9orf72* status, site of onset and gender (DIC: 42,485) than the model including *C9orf72* status only (DIC: 42,646). Figure 2 shows distinct curves between *C9orf72* normal patients and *C9orf72* expanded patients in males only, while in females the trends are virtually indistinguishable. Among males there appears to be a greater distinction by *C9orf72* status in spinal onset patients than in bulbar onset patients.

**Figure 3.**
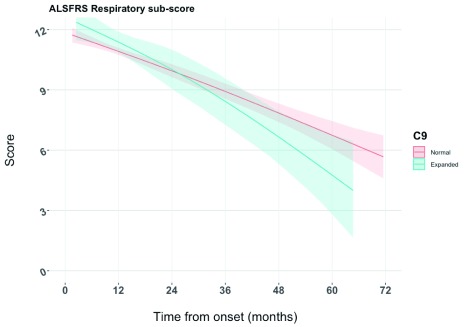
Predicted Amyotrophic Lateral Sclerosis Functional Rating Scale-revised (ALSFRS-R) respiratory score by C9orf72 status generated from joint longitudinal and time to event model.

Of the 630 patients included in the study, only 450 had a total of 1,728 contemporaneous SNIP and ALSFRS-R __resp_ measurements. Figure 3 displays the longitudinal characteristics of the ALSFRS-R __resp_ by *C9orf72* expansion status. In contrast to Figure 1, Figure 3 shows that the ALSFRS-R __resp_ is indistinguishable between *C9orf72* normal and *C9orf72* expanded patients in the earlier years of the disease course. After approximately three years, the trends begin to diverge; however, the credible intervals remain overlapping.

## Discussion

In this study we confirmed the findings of Miltenberger-Miltenyi *et al*. ^16^ that carriage of the *C9orf72* expansion in ALS is associated with both survival and an accelerated decline in respiratory function in comparison to ALS without the expansion. We also found that the ALSFRS-R __resp_ did not differentiate rate of decline by *C9orf72* status within the first three years, as shown by direct respiratory strength testing using occluded SNIP. This confirms the similar finding using %FVC to measure respiratory function by Miltenberger-Miltenyi *et al*. ^16^. Our analysis differs from that of Miltenberger-Miltenyi *et al.* ^16^ ** through the use of splines to allow for non-linear trends for SNIP and ALSFRS-R __resp_ decline over time, and the use of joint models to account for differential loss to follow-up and estimation of the effect of the longitudinal terms in the survival sub-model. Additionally, our exploratory model demonstrated that the association between respiratory function and *C9orf72* status is more distinct in male patients, particularly in those with spinal onset disease, which may explain our previous observations in European populations of a worse prognosis in male spinal onset *C9orf72* expansion carrying patients ^13^.

The comparison of hazard ratios from Cox and joint models indicates that after controlling for longitudinal SNIP measurements, the bulbar onset and presence of the *C9orf72* expansion are no longer strongly associated with the risk of death, and that the SNIP measurement itself is predictive of survival. These results are suggestive that respiratory strength decline may explain part of the survival effect of the *C9orf72* expansion in ALS. Alternatively, another characteristic of *C9orf72* expansion ALS, such as cognitive or behavioural dysfunction, may mediate this relationship ^5, 12, 25^. Conversely, it is plausible that both disease subtypes follow a common path towards respiratory dysfunction after some earlier biochemical convergence, with the *C9orf72* expanded group having reached that point more rapidly - i.e. the findings may reflect a faster overall disease process rather than faster progress in respiratory function alone. Therefore, while Miltenberger-Miltenyi *et al.* hypothesise that a pathophysiological link between *C9orf72* and respiratory function may occur due to an interaction between disordered regulation of the homeobox gene (Hoxa5) and *C9orf72* mutated proteins ^16, 26^, such direct interaction between *C9orf72* and respiratory function may not be required to explain these results.

Our finding that the ALSFRS-R __resp_ did not differentiate between *C9orf72* normal and *C9orf72* expanded ALS is congruent with findings that ALSFRS-R __resp_ questions are not sensitive to the respiratory burden of the majority of patients ^27– 29^. Our previous study on longitudinal sub-scores of the ALSFRS-R in ALS cases unstratified by *C9orf72* status found that ALSFRS-R __resp **_had a worse ability to distinguish spinal and bulbar onset disease than bulbar and motor sub-scores did, and additionally had less prognostic value ^27^. In addition, Franchignoni *et al.* found that the ALSFRS-R __resp_ questions were subject to frequent ceiling responses and suggested the addition of one or two questions of intermediate difficulty to improve reliability and personal discrimination of this sub-score ^29^. Therefore, our current results suggest that the occluded SNIP could provide a suitable metric with which to augment the ALSFRS-R as an alternative to additional intermediate questions. Furthermore, as joint models provide a framework for combined analysis of longitudinal measurements and survival, they can extend to model multiple longitudinal measurements, and in addition, recent analysis has shown that joint models may provide greater statistical power in ALS trials with functional and mortality outcomes compared to other approaches ^30^.

Our analysis benefits from a large number of longitudinal measurements with up to six years follow-up in a cohort of 630 ALS patients, including 58 who carried the *C9orf72* expansion. Even though we used the occluded SNIP as a metric of respiratory function, which differs from the use of %FVC by Miltenberger-Miltenyi *et al*. ^16^, our results are congruent with theirs. In addition, the use of joint models allowed us to demonstrate the impact of longitudinal respiratory function on survival while at the same time accounting for differential loss to follow-up in longitudinal occluded SNIP measurements. The main limitation is that the analysis did not include longitudinal data on cognitive or behavioural function, which may play an important role in mediating the effects of *C9orf72* on survival in ALS.

## Conclusions

Our results confirm findings from Portugal that the *C9orf72* repeat expansion is associated with both survival and accelerated respiratory function decline in ALS, and that the ALSFRS-R __resp_ does not differentiate respiratory function between *C9orf72* normal and *C9orf72* expanded cases in the first three years of follow-up. Furthermore, we demonstrated through the use of joint models that respiratory function measured using occluded SNIP carries prognostic importance and may explain previous observations in European cohorts of a worse prognosis in male spinal onset ALS patients that carry the *C9orf72* expansion.

## Data availability

The raw data from this study cannot be sufficiently de-identified, and therefore are not publicly available. As ALS is a rare disease, we are very conscious of protecting privacy of patients. In this particular analysis, low numbers of cases at certain age ranges mean we could not guarantee privacy if we were to publish the data in full. However, the data from the current study are available for further research purposes on reasonable request. To access the data, please contact the Principal Investigator ( orla@hardiman.net). Researchers must provide a written proposal on how the data will be used in research before access is granted.

## Software availability

Source code available from: https://github.com/jpkrooney/ALS_C9orf72_Resp_function_Paper

Archived source code at time of publication: https://doi.org/10.5281/zenodo.3445433

License: GPL3
