# Supplementary material for: The C9orf72 expansion is associated with accelerated respiratory function decline in a large Amyotrophic Lateral Sclerosis cohort
Source: HRB Open Res. 2019 Sep 26;2:23. [Version 1] doi: 10.12688/hrbopenres.12940.1 (PMC7140774; doi:10.12688/hrbopenres.12940.1)
Supplement: Peer review history

# Peer review: Reviewer response for version 1

**DOI:** 10.21956/hrbopenres.14020.r27111

**Published:** 2020-03-10

**Authors:** Christian Lunetta

**Affiliations:** 1 NEuroMuscular Omnicentre (NEMO), Fondazione Serena Onlus, Milan, Italy

The study aimed to evaluate the respiratory function decline in ALS patients according the genetic background, in particular, regarding the C9ORF expansion. The study confirmed that patients carrying C9ROF72 expansion were associated with a more rapid decline compared than those without the C9ORF72 expansion. Moreover, male C9ORF72 patients were associated with a more rapid decline compared to those without the C9ORF72 expansion.

 The study is well-written and the population included is a large number of patients.

 A minor concern, no data about the cognitive function are included in the description of the patients. Taking into account the frequent associaton of C9ORF72 ALS with fronto-temporal dysfunction, it could be interesting to understand if the cognitive function could negatively affect the respiratory evaluation. In other words, if this data are available, the reviewer suggests to correct the result for the cognitive function.

I confirm that I have read this submission and believe that I have an appropriate level of expertise to confirm that it is of an acceptable scientific standard.

# Peer review: Reviewer response for version 1

**DOI:** 10.21956/hrbopenres.14020.r26889

**Published:** 2019-11-11

**Authors:** Gabriel Miltenberger Miltényi, Mamede de Carvalho

**Affiliations:** 1 Physiology Institute, Faculty of Medicine, Instituto de Medicina Molecular, University of Lisbon, Lisbon, Portugal

The authors demonstrated in a large ALS patient group that the *C9orf72* expansion is a risk factor for faster decline of the respiratory function and for shorter survival. The work is in concordance with a previous study on Portuguese patients, but it is using another method for respiratory function measurement and - partially - different statistical tools. 

 The present study was well-designed and it helps to improve the knowledge of possible risk factors in ALS.

 We have some minor comments:

1. As the present work, using sniff nasal inspiratory pressure (SNIP, an inspiratory test) refers to the results of another similar study that used forced vital capacity (FVC, a global test depending on inspiratory and expiratory strength) for measuring the respiratory function in ALS, it might be helpful to shortly compare these two methods. For example, significant correlations have been found between both FVC and SNIP, and the amplitude of the motor response of the phrenic nerve in ALS (Pinto *et al*., 2016 ^1^; Fantini *et al*., 2016 ^2^; Noda *et al*., 2016 ^3^).
  This might be even more important as both studies (the present work and that of Portuguese patients) demonstrated that the respiratory subscore of ALS-FRS-R, on the contrary, did not show correlation with the *C9orf72* status nor with the respiratory decline.
2. The authors mention that the main limitation of their study was that their analysis did not include longitudinal data on cognitive or behavioural function, but they are not mentioning the reason for this. Also, the baseline assessment (presented in Table 1) did not include data on cognitive or behavioural function.
3. For both studies, this one and the one from Portugal, the role of cognitive decline associated with *C9orf72 *mutation on the collaboration required in the involved volitional tests to assess respiratory function (SNIP and FVC), remains unclear. This topic could deserve some discussion.

As a summary, we think that the present work is important for the better understanding of the pathophysiology of ALS.

We confirm that we have read this submission and believe that we have an appropriate level of expertise to confirm that it is of an acceptable scientific standard.

## References

[1] : Ultrasound for assessment of diaphragm in ALS. *Clin Neurophysiol*.2016;127(1) : 10.1016/j.clinph.2015.03.024 892-897 10.1016/j.clinph.2015.03.024 25971723

[2] : Ultrasound assessment of diaphragmatic function in patients with amyotrophic lateral sclerosis. *Respirology*.2016;21(5) : 10.1111/resp.12759 932-8 10.1111/resp.12759 26994409

[3] : Ultrasonographic diaphragm thickness correlates with compound muscle action potential amplitude and forced vital capacity. *Muscle Nerve*.2016;53(4) : 10.1002/mus.24902 522-7 10.1002/mus.24902 26357011
